# In-situ and invasive carcinoma within a phyllodes tumor associated with lymph node metastases

**DOI:** 10.1186/1477-7819-2-46

**Published:** 2004-12-15

**Authors:** Jeremy R Parfitt, Chris Armstrong, Frances O'Malley, Joan Ross, Alan B Tuck

**Affiliations:** 1 Department of Pathology, London Health Sciences Centre, University of Western Ontario, London, Ontario, Canada; 2 Department of Pathology and Laboratory Medicine, Mount Sinai Hospital and University of Toronto, Toronto, Ontario, Canada; 3 Lambton Hospital Group – St. Joseph's, Sarnia, Ontario, Canada

## Abstract

**Background:**

Phyllodes tumors (cystosarcoma phyllodes) are uncommon lesions in the female breast. Rarely, the occurrence of carcinoma within a phyllodes tumor has been reported in the literature, but has never been associated with lymph node metastases.

**Case presentation:**

A 26-year-old woman presented with a firm, mobile, non-tender mass in the left breast and palpable lymph nodes in the left axilla. The excised lesion appeared well circumscribed and lobulated, with variable fleshy and firm areas. Microscopic examination showed a circumscribed fibroepithelial lesion with a well developed leaf-like architecture, in keeping with a benign phyllodes tumor. The epithelial component showed extensive high grade ductal carcinoma *in-situ *(DCIS) and invasive carcinoma of no special type, located entirely within the phyllodes tumor. Subsequent axillary lymph node dissection revealed metastatic carcinoma in four lymph nodes.

**Conclusions:**

Although rare, phyllodes tumors may harbor DCIS and invasive carcinoma, with potential for lymph node metastasis.

## Background

Phyllodes tumors constitute less than 1% of breast tumors and 2–3% of fibroepithelial breast tumors [1]. They usually occur in middle-aged to elderly women but can occur at any age. No single feature is reliable in predicting clinical behavior of phyllodes tumors. Several histological parameters should be evaluated, including stromal cellularity, atypia, mitoses, stromal overgrowth, infiltrative borders, and presence or absence of necrosis [1,2]. A mitotic rate of less than 5 per 10 high power field (HPF) suggests benign behavior, while a mitotic figure rate of more than 10 per 10 HPF suggests malignant potential [2,3]. Most of these tumors are benign, but up to 30% show malignant stroma [4]. Metastases, usually hematogenous rather than lymphatic, have been reported to occur at a rate of 13% in 10 years for malignant phyllodes tumors [4]. As malignant phyllodes tumors usually spread by a hematogenous rather than a lymphatic route, axillary lymph node dissection is generally not recommended. Importantly, local recurrences are common even in benign tumors and are seen in up to 8% in 10 years [4].

We describe a case of *in-situ *and invasive carcinoma occurring within a benign phyllodes tumor in a 26-year-old woman, which, to our knowledge, is the youngest presenting age for this combination tumor. We also report the first case of this combination tumor with documented lymph node metastases. Our case highlights the importance of assessing phyllodes tumors for concurrent carcinomatous involvement, as this may affect both the need for axillary lymph node examination and subsequent treatment options.

## Case presentation

A 26-year-old woman presented with a 1 month history of a palpable mass in her left breast. Her mother died at the age of 31 years of breast cancer. Examination revealed a 2 cm firm and mobile mass in the lower outer quadrant of the left breast. The left axilla contained two enlarged palpable lymph nodes.

Mammography showed a 2 cm oval density that was homogenous with sharp margins. Ultrasound characterized the mass as solid but with a complex internal architecture. Fine needle aspiration of the mass was performed. It was positive for carcinoma, showing a very cellular sample with many malignant epithelial cells in cohesive groups, as well as lying singly in a background of necrosis and inflammation. The surgeon subsequently performed an excisional biopsy.

**Figure 1:**
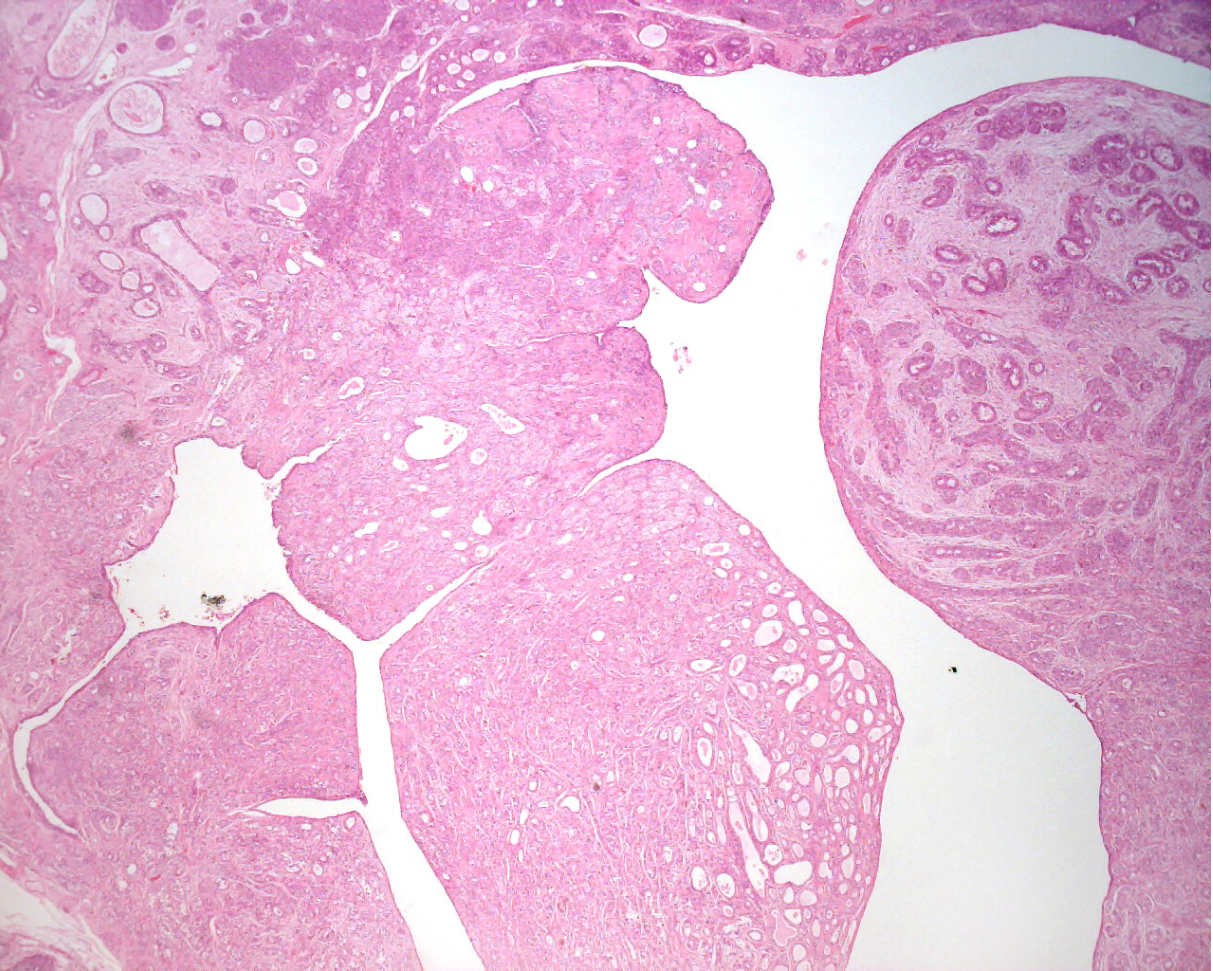
Low power photomicrograph of the phyllodes tumor: Histologically, the neoplasm was biphasic, with proliferating stroma, compressed glands, and a well developed leaf-like architecture (H&E, original magnification 40×).

**Figure 2:**
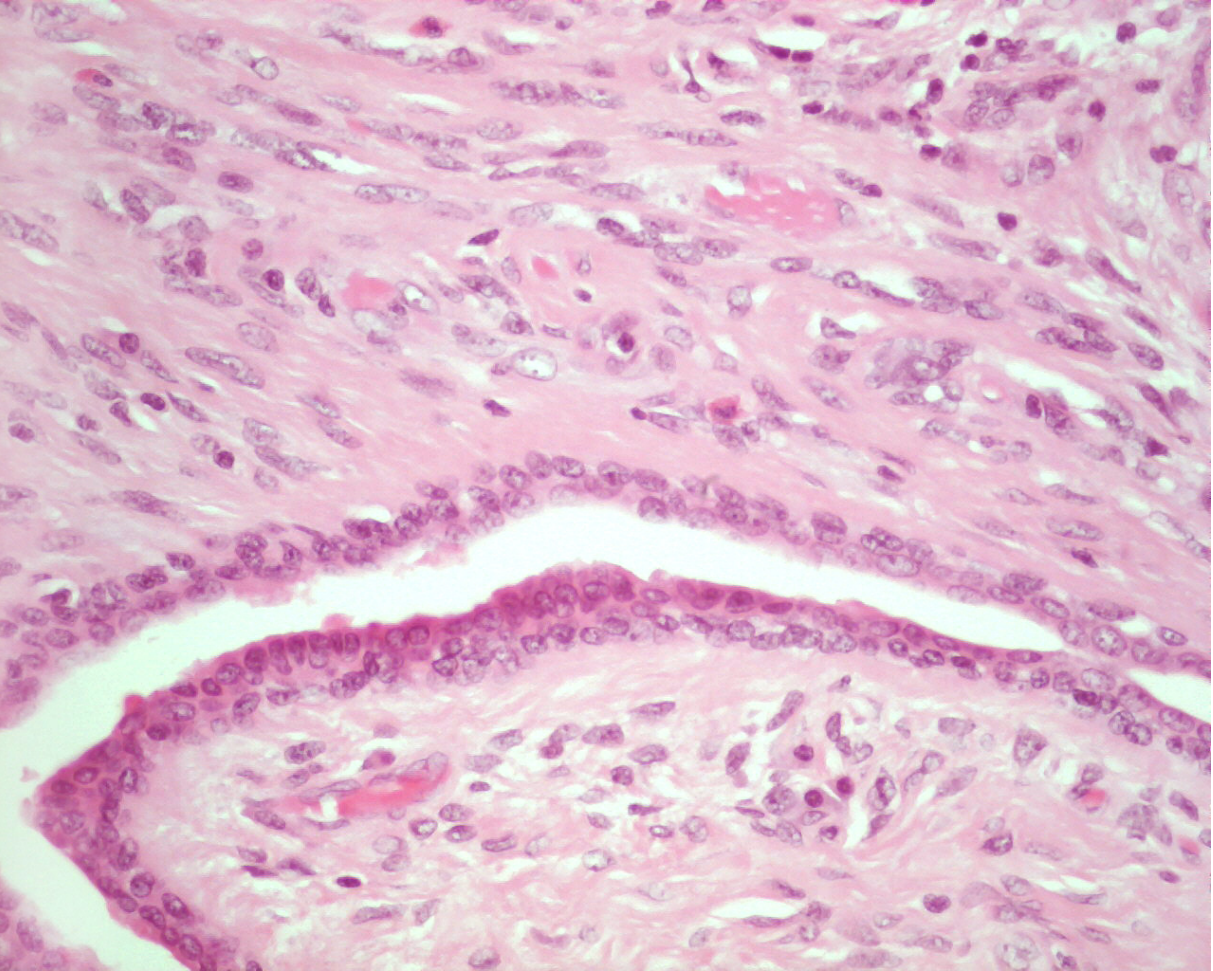
High power photomicrograph of the phyllodes tumor: The stroma lacked cellular atypia and mitoses, consistent with a benign phyllodes tumor (H&E, original magnification 400×).

**Figure 3:**
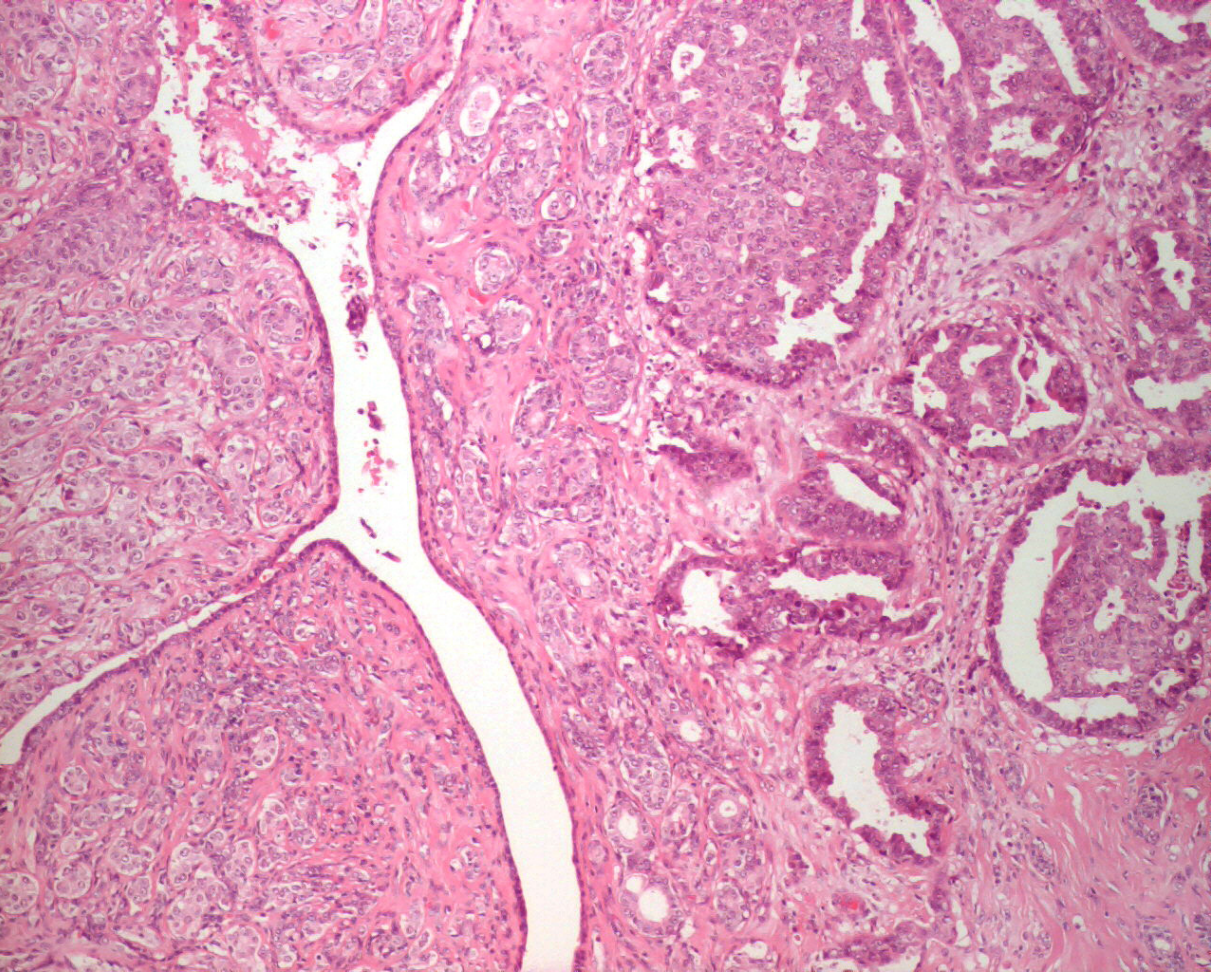
Photomicrograph of the carcinomatous component: The epithelial component showed DCIS and invasive mammary carcinoma (H&E, original magnification 100×).

**Figure 4:**
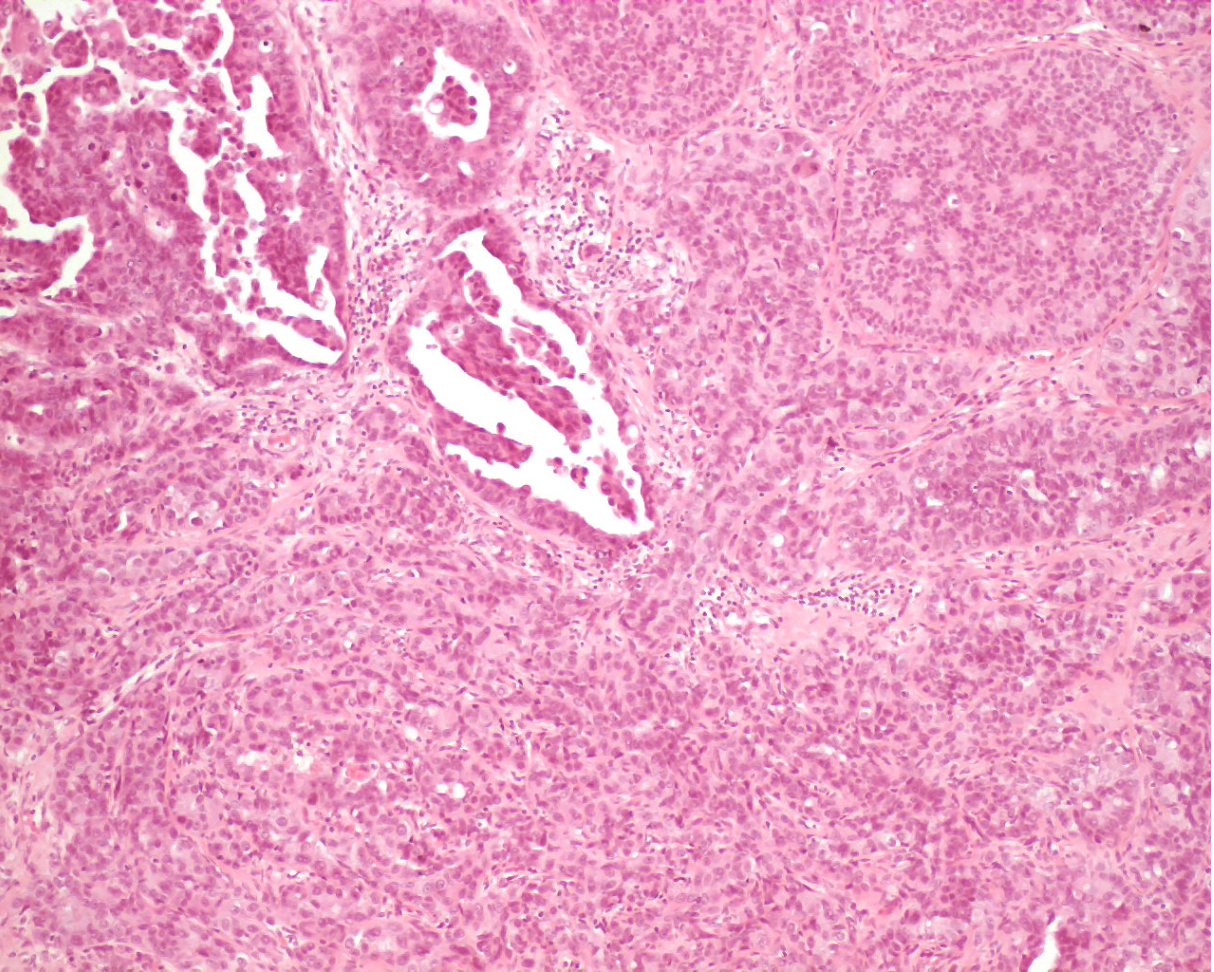
Photomicrograph of the DCIS patterns: The DCIS showed cribriform and micropapillary architecture, with necrosis (H&E, original magnification 100×).

Grossly, the excised mass measured 3.3 cm × 2.3 cm × 2.0 cm and appeared well circumscribed. The cut surface was grey and lobulated with variable fleshy and firm areas. Histological examination revealed a well-circumscribed biphasic neoplasm, consisting of proliferating stroma and compressed glands, displaying a well developed leaf-like architecture. The stroma was moderately cellular, mildly vascular, and lacked cytological atypia, mitoses, and stromal overgrowth. The features were consistent with a benign phyllodes tumor (figures 1 &2). More importantly, the epithelial component of the phyllodes tumor showed extensive ductal carcinoma *in-situ*, with multiple foci of invasive mammary carcinoma (figure 3). The DCIS was of high nuclear grade, cribriform and micropapillary architecture, with (comedo-type) necrosis (figure 4). The invasive carcinoma was of no special type, SBR Grade III/III (Score 8/9). Multiple tumor emboli were present within endothelial-lined spaces adjacent to the tumor, but the surrounding breast was otherwise unremarkable. Although the carcinomatous component was confined to the phyllodes tumor, the lesion was <1 mm from the nearest resection margin. Immunohistochemistry using smooth muscle myosin heavy chain and muscle specific actin demonstrated absence of the myoepithelial cell layer within areas of invasive carcinoma, and focal loss within areas of DCIS. There was intensely positive staining within 80% of tumor cells for estrogen and progesterone receptors.

The patient went on to have a left axillary lymph node dissection and subsequent pathological examination revealed 4 of 13 nodes positive for metastatic adenocarcinoma. She received adjuvant CEF (Cyclophosphamide, Epirubicin, 5-Fluorouracil) chemotherapy and underwent further local excision to achieve wider margins; pathological examination was negative for residual tumor. She subsequently received local radiation and is currently undergoing tamoxifen therapy. The patient is presently alive and disease free, nearly three years after presentation.

## Discussion

The epithelial component of phyllodes tumors (whether benign or malignant) may show a range of metaplastic (apocrine, squamous) and proliferative changes [5,6]. However, carcinoma arising within a phyllodes tumor is decidedly rare, with less than 30 reported cases in the literature. The age of the previously reported patients with coexistent carcinoma and phyllodes tumor ranged from 31–80 years, with most of the patients in the 5th or 6th decades [7,8]. This case represents the youngest age at presentation with this combination tumor. The reported subtypes include *in-situ *and invasive lobular and ductal (no special type) carcinoma, tubular carcinoma and squamous carcinoma [2-7,9-13]. Some authors have noticed greater atypia in epithelial components of recurrent phyllodes tumors. Squamous carcinoma, typically rare in the breast, seems to occur more often in phyllodes tumors, compared to patients without phyllodes tumors [10].

The prognosis for cases of carcinoma within phyllodes tumors is generally favorable, with no deaths yet reported [9,10]. The stroma is benign in the majority and cancer detection often occurs early because the patient presents with a rapidly enlarging mass [10]. Our case appears to represent the first documentation of lymph node spread of the carcinomatous component from within a phyllodes tumor. Axillary lymph node dissection is not part of the standard treatment for phyllodes tumors as lymph node spread is rare [14]. Several authors have suggested treating carcinoma within phyllodes tumors according to the carcinomatous component [9,14]. Our case supports this view, particularly with respect to the need for assessment of axillary lymph node status in determining prognosis and treatment.

## Conclusions

Invasive carcinoma occurring within a phyllodes tumor is rare; occurrence of axillary lymph node metastasis is even rarer. Besides the stromal component, the epithelial component of phyllodes tumors should also be examined in detail, as it may harbor an *in situ*or invasive carcinoma.

## Competing Interests

The authors declare that they have no competing interests.

## Authors' Contributions

**JRP **prepared the manuscript.

**CA **and **ABT **were involved with initial diagnosis of the pathologic specimen and were involved with manuscript editing.

**FO **provided consultation for the final diagnostic interpretation and was involved with manuscript editing.

**JR **provided patient history, obtained patient consent and helped with manuscript editing.

All authors read and approved the final manuscript.

## References

[B1] Rosen PP, Oberman HA, Rosai J, Sobin LH (1993). Cystosarcoma phyllodes. Atlas of tumor pathology. Tumors of the mammary gland.

[B2] Pandey M, Mathew A, Kattoor J, Abraham EK, Mathew BS, Rajan B, Nair KM (2001). Malignant phyllodes tumor. Breast J.

[B3] Padmanabhan V, Dahlstrom JE, Chong GC, Bennett G (1997). Phyllodes tumor with lobular carcinoma in situ and liposarcomatous stroma. Pathology.

[B4] Chaney AW, Pollack A, McNeese MD, Zagars GK, Pisters PW, Pollock RE, Hunt KK (2000). Primary treatment of cystosarcoma phyllodes of the breast. Cancer.

[B5] Leong ASY, Meredith DJ (1980). Tubular carcinoma developing within a recurring cystosarcoma phyllodes of the breast. Cancer.

[B6] Naresh KN (1997). Cancerization of phyllodes tumor. Histopathology.

[B7] Knudsen PJT, Ostergaard J (1987). Cystosarcoma phyllodes with lobular and ductal carcinoma in situ. Arch Pathol Lab Med.

[B8] Nishimura R, Takahiro H, Imoto S, Mukai K (1998). Malignant phyllodes tumor with a noninvasive ductal carcinoma component. Virchows Arch.

[B9] De Rosa G, Ferrara G, Goglia P, Ghicas C, Zeppa P (1989). In situ and microinvasive carcinoma with squamoid differentiation arising in a phyllodes tumor: report of a case. Tumori.

[B10] Grove A, Kristensen LD (1986). Intraductal carcinoma within a phyllodes tumor of the breast: a case report. Tumori.

[B11] Schwickerath J, Blessing MH, Wolff F (1992). Seltene Erscheinungsform eines kombinationstumors aus cystosarcoma phylloides malignum und eines intraductalen karzinoms. Geburtsh U Frauenheilk.

[B12] Gebrim LH, Bernardes Junior JR, Nazario AC, Kemp C, Lima GR (2000). Malignant phyllodes tumor in the right breast and invasive lobular carcinoma within fibroadenoma in the other: case report. Sao Paulo Med J.

[B13] Kodama T, Kameyama K, Mukai M, Sugiura H, Ikeda T, Okada Y (2003). Invasive lobular carcinoma arising in phyllodes tumor of the breast. Virchows Arch.

[B14] Ishida T, Izuo M, Tadakazu K (1984). Breast carcinoma arising in cystosarcoma phyllodes: report of a case with a review of the literature. Jpn J Clin Oncol.

